# Psychosocial and spiritual correlates of migration intentions among nursing and midwifery students: a descriptive correlational study

**DOI:** 10.1186/s12912-026-04414-4

**Published:** 2026-03-06

**Authors:** Emine Karacan, Soner Berşe, Emine Karacan, Zeynep Güngörmüş

**Affiliations:** 1 Department of Health Care Services, Health Services Vocational School, Elderly Care Program, Gaziantep Islamic Science and Technology University, Gaziantep, Türkiye; 2 Division of Nursing Fundamentals, Department of Nursing, Faculty of Health Sciences, , Gaziantep University, Gaziantep, Türkiye; 3 Department of Medical Services and Techniques, Dörtyol Health Services Vocational School, Medical Documentation and Secretarial Program, İskenderun Technical University, Hatay, Türkiye; 4 Department of Nursing, Faculty of Health Sciences, Gaziantep Islamic Science and Technology University, Gaziantep, Türkiye

**Keywords:** Brain drain, Future expectations, Hopelessness, Religious belief, Nursing, Midwifery

## Abstract

**Introduction:**

The migration of highly educated individuals, particularly in healthcare, from developing to developed countries has increased in recent years. Nurses and midwives seeking better living and career opportunities abroad pose challenges to the quality and sustainability of healthcare services. Understanding the migration intentions of nursing and midwifery students is essential for anticipating future workforce issues and guiding effective policy responses.

**Aim:**

To examine the relationships between nursing and midwifery students’ future expectations, levels of hopelessness, religious belief levels, and migration inclinations.

**Method:**

This descriptive and correlational study was conducted among nursing and midwifery students (*n* = 480) at the Faculty of Health Sciences at Gaziantep University, Türkiye. Data were collected through face-to-face interviews using the Descriptive Characteristics Form, the Future Expectations Scale for Adolescents (FESA), the Beck Hopelessness Scale (BHS), the Religious Belief Scale for Adolescents (RBSA), and the Attitudes Scale for Brain Drain Among Nursing Students (ASBD). Data were analyzed using SPSS version 24.

**Results:**

Among the participants, 59.4% were aged between 18 and 20 years, 79.2% were female, and 95.6% were Turkish nationals. A total of 43.8% reported considering living abroad, and 26.9% expressed a preference for Germany. Economic and professional factors were the primary motivations for 40.6% of students considering migration. It was determined that students’ future expectations (mean FESA score, 91.65 ± 39.31) and levels of hopelessness (mean BHS score, 10.90 ± 4.96) were moderate, their level of religious belief (mean RBSA score, 82.24 ± 12.15) was high, and their tendency to migrate (mean ASBD score, 28.31 ± 14.01) was low.

**Conclusion:**

As a result, it was determined that a significant portion of nursing and midwifery students considered migrating abroad, most often choosing Germany. Their intention to migrate stemmed primarily from economic and professional factors. The findings also indicate that students’ future expectations and hopelessness levels were moderate, their religious belief levels were high, and their overall tendency to migrate was low. As religious belief levels increased, students’ future expectations increased, their hopelessness decreased, and their tendency to migrate decreased. Regression analyses revealed that future expectations and religious belief were depressing, while hopelessness increased, and these three variables, combined, played a strong predictive role in explaining students’ migration intentions.

**Clinical trail number:**

Not applicable.

## Highlights

Religious belief was the strongest protective factor against students’ migration intentions.

Higher future expectations were associated with lower levels of hopelessness and migration tendency.

Students with greater satisfaction in their education and profession showed less desire to migrate.

Despite high reported interest in migration, overall migration tendencies remained moderate.

A holistic approach addressing psychosocial and spiritual well-being may reduce migration intentions among healthcare students.

## Introduction

The term “brain drain” refers to the migration of highly educated and skilled professionals from developing to developed countries that offer better living conditions and employment opportunities [1]. In recent years, this phenomenon has become one of the most rapidly expanding forms of international migration [2, 3]. The healthcare sector is among the most significantly affected sectors [4], with migration being particularly prominent in critical fields such as nursing and midwifery [3, 5]. A recent study in Türkiye reported that 76.3% of nurses expressed a desire to work abroad [6]. According to the World Health Organization (WHO), there are currently 29 million nurses and 2.2 million midwives worldwide, and by 2030, an additional 4.5 million nurses and 300,000 midwives will be needed globally [7, 8]. Furthermore, OECD data indicate that the number of nurses per 1,000 people in Turkey will be only 2.9 in 2023, which is well below the average of developed countries [9]. The prospect of higher salaries, improved working conditions, greater opportunities for education, career advancement, and job satisfaction in developed countries are major factors driving migration among nurses and midwives [3, 10].

Structural deficiencies within the healthcare systems of developing countries, such as excessive workloads, exposure to psychological and physical violence, political instability, and low wages, adversely affect healthcare professionals’ motivation and contribute to the ongoing brain drain [10]. The migration of nurses and midwives weakens national health systems, compromises the accessibility and sustainability of healthcare services, and results in a significant decline in the quality of care [11]. Furthermore, this exodus poses substantial challenges to the delivery of essential healthcare services and impedes the advancement of innovative practices and effective health policies [12]. Globalization, economic crises, changes in educational systems, and broader social transformations increasingly shape the future expectations, levels of hopelessness, and religious beliefs of younger generations, particularly those studying in critical fields such as health sciences [1, 13]. These factors contribute to a growing tendency among nursing students to consider emigration as a viable option [1, 14].

Future expectations refer to how individuals envision their aspirations, goals, and role within society [15, 16]. For nursing and midwifery students, these expectations are shaped by the emotional experiences they encounter during their education, which play a key role in the formation of their professional identities. Hopelessness is defined as a motivational tendency to give up, marked by diminished optimism and a pattern of negative expectations about one’s self and future circumstances [17]. Such feelings can significantly influence behavior and decision-making, including the consideration of employment abroad [1, 14, 18]. Religious beliefs also play a significant role in shaping students’ psychological well-being, resilience, and problem-solving capacity [19–22]. Strong religious convictions can anchor students to moral values and public service, potentially reducing their inclination to migrate, while for some, the aspiration to freely practice religion in more tolerant settings may act as a pull factor [23–25].

Students’ future expectations, levels of hopelessness, and religious beliefs, combined with economic and professional aspirations, represent key psychological factors influencing migration tendencies. While previous research on brain drain has focused mainly on economic and career-related factors, the role of religious beliefs remains underexplored. The present study aims to investigate the relationships among future expectations, hopelessness, religious beliefs, and the intention to migrate to developed countries among nursing and midwifery students in Türkiye, addressing a gap in the literature regarding how religious beliefs affect migration tendencies.

*Research Question*

1. Is there a difference between the socio-demographic characteristics (e.g., age, gender, educational level) of nursing and midwifery students and their future expectations, hopelessness, religious belief levels, and tendency towards brain drain?
2. To what extent do nursing and midwifery students’ future expectations, hopelessness, and religious belief levels predict brain drain?

## Materials and methods

### Study design

This study employed a descriptive and correlational design.

### Study setting and duration

The study was conducted between December 2024 and May 2025 at the Departments of Nursing and Midwifery, Faculty of Health Sciences, Gaziantep University. Data collection was carried out between December 2024 and January 2025.

### Study population and sample

The study population consisted of undergraduate students enrolled in the nursing (n = 1024) and midwifery (n = 350) departments of the Faculty of Health Sciences at Gaziantep University. The sample size was calculated using the G*Power software, based on the study titled “ Determination The Factors Influenced The Hopelessness Level Of Nursing Students (2020)” [26]. A sample size of 340 students was required to detect an effect size of d = 0.392 with 95% power (1-β = 0.95) and a significance level of α = 0.05 for a two-tailed test. The minimum sample size was found to be 340 students. To strengthen and generalize the research results, 480 students (421 nursing and 59 midwifery) were reached in our study.

*Inclusion criteria for the study were*:

1. Being enrolled in the nursing or midwifery department of Gaziantep University’s Faculty of Health Sciences,
2. Being a student during the study period,
3. Agreeing to participate voluntarily in the study.

*Exclusion criteria for the study were*:

1. Incomplete survey form.

### Data collection tools

#### Descriptive characteristics form

This form, developed by the authors, consisted of 21 questions, including 16 questions addressing students’ socio-demographic characteristics (e.g., age, gender, income level, marital status) and 5 questions assessing intentions to migrate.

#### Future expectations scale (FES)

Originally developed by McWhirter and McWhirter (2008) [27], the FESA was adapted into Turkish by Tuncer (2011). It contains 25 items rated on a 7-point Likert scale (1 = Strongly Disagree, 7 = Strongly Agree). The scale comprises four subdimensions: “Work and Education” (Items 1–11), “Marriage and Family” (Items 12–18), “Religion and Community” (Items 19–21), and “Health and Life” (Items 22–25). Possible total scores range from 25 to 175, with higher scores indicating more positive and stronger expectations for the future. The Cronbach’s alpha coefficient for the Turkish version of the FESA scale was 0.92, as reported by Tuncer (2011) [28]. In the present study, the Cronbach’s alpha was also 0.92.

#### Beck hopelessness scale (BHS)

Developed by Beck et al., the BHS is a self-report measure of one’s negative expectations regarding the future [29]. Reliability and validity of a Turkish version of the BHS were demonstrated by Durak and Palabıyıkoğlu (1994). The BHS consists of 20 true-false statements of which 9 are keyed false and 11 are keyed true. Specifically, items 2, 4, 7, 9, 11, 12, 14, 16, 17, 18, and 20 are assigned a score of 1 if the response is “true”, while items 1, 3, 5, 6, 8, 10, 13, 15, and 19 are assigned a score of 1 if the response if “false”. Otherwise, they are scored 0 points. The BHS assess three aspects of hopelessness: Feelings about the future (Items 1, 6, 13,15, 19), Loss of motivation(2, 3, 9, 11, 12, 16, 17, 20), and Expectations about the future (Items 4,7,8,14,18). The total score can range from 0 to 20, with higher scores reflecting higher levels of hopelessness. Durak and Palabıyıkoğlu (1994) reported a Cronbach’s alpha coefficient of 0.85 [30]. In the present study, Cronbach’s alpha values for the subdimensions and total scale were 0.81 (Feelings about the future), 0.79 (Loss of motivation), 0.60 (Expectations about the future), and 0.87 (total).

#### Religious belief scale (RBS)

The RBSA was developed and validated by Horozcu and Demir (2018) [31]. The scale consists of 18 items, has a unidimensional structure, and is rated on a 5-point Likert scale (1 = I never think this way, 5 = I always think this way). Items 5, 6, 10, 12, 16, 17, and 18 are reverse scored. The total score ranges from 18 to 90. Higher scores reflect stronger belief in Islam and a less religious doubt. The Cronbach’s alpha coefficient of the RBSA was reported as 0.93 by Horozcu and Demir (2018), and was also found to be 0.93 in the current study.

#### Attitudes scale for brain drain among nursing students (ASBD)

The ASBD was developed by Öncü et al. (2018) to assess nursing students’ attitudes toward emigration [2]. The scale consists of 16 items and uses a 5-point Likert format. It has a unidimensional structure with two components: Pull Factors (items 1, 2, 3, 4, 5, 6, 8, 10, 12, 14, 15, and 16) and Push Factors (items 7, 9, 11, and 13). Items 3 and 15 are reverse scored. Total possible scores range from 16 to 80, with higher scores indicating a stronger tendency toward migration. The Cronbach’s alpha coefficients reported by the original developers were 0.91 for the full scale, 0.88 for the “pull factors” subdimension, and 0.86 for the “push factors” subdimension [2]. In the current study, Cronbach’s alpha values were 0.90 for pull factors, 0.93 for push factors, and 0.93 for the total scale.

### Data collection

Data were collected through face-to-face interviews using the specified data collection tools.

#### Data analysis

Data obtained from the study were analyzed using IBM SPSS Statistics 24.0 (IBM Corp., Armonk, NY, USA). Categorical variables were presented with frequency and percentage distributions, while numerical variables were presented with descriptive statistics such as mean and standard deviation. The data were assessed for normal distribution using the Kolmogorov-Smirnov test, along with Skewness and Kurtosis values. In the current study, Skewness values ​​ranged from − 1.984 to 0.994, and Kurtosis values ​​ranged from − 1.052 to 1.889. In the literature, data are considered normally distributed if Skewness and Kurtosis values ​​range from − 2 to + 2 [32]. Additionally, due to a sample size exceeding 50, the Kolmogorov-Smirnov test was used to assess normality [33], and p values ​​>0.05 indicated normal distribution. For normally distributed data, differences between two groups were examined using the independent samples t-test, and differences between more than two groups were examined using one-way analysis of variance (one-way ANOVA). Homegeneity of variances in ANOVA tests was assessed using the Levene test. Hochberg’s GT2 post-hoc test was applied when variances were homogeneous, and Games-Howell post-hoc test was applied based on the Welch ANOVA results when variances were not homogeneous. In addition, the relationships between the total scores and subscales of the scales were examined using Pearson correlation analysis. To assess the effects of the independent variables on ASBD, multiple linear regression analysis was conducted, with all independent variables entered into the model simultaneously using the Enter (Forced Entry) method. This approach allows for the evaluation of each variable’s unique contribution to ASBD while controlling for the effects of the other variables. All analyses were interpreted at a 95% confidence level and a significance level of *p* < 0.05.

### Ethical considerations

Ethical approval for the study was obtained from the Institutional Review Board of Gaziantep Islam Science and Technology University (Protocol No: 2024/454, Decision No: 454.40.04, Date: 25.11.2024). Institutional permission was also secured from the public university where the research was conducted. Informed consent was obtained from all participating students. The study was conducted in accordance with the principles laid out in the Declaration of Helsinki. 

## Results

The descriptive characteristics of nursing and midwifery students are presented in Table 1.

**Table 1:** Comparison of total scores on the future expectations scale for adolescents (FESA), Beck hopelessness scale (BHS), religious belief scale for adolescents (RBSA), and attitudes scale for brain drain among nursing students (ASBD) according to the. Descriptive characteristics of nursing and midwifery students

Descriptive Characteristics (*n* = 480)		*n* (%)	FESA Total score	BHS Total score	RBSA Total score	ASBD Total score
**Age group**,** years**	18-20^a^	285 (59.4)	89.28 ± 39.76	6.56 ± 4.70	83.56 ± 10.38	55.28 ± 14.26
	21-24^b^	188 (39.2)	95.33 ± 38.64	7.30 ± 5.29	80.65 ± 14.00	56.92 ± 13.76
	25 or older^c^	7 (1.5)	89.28 ± 35.82	10.42 ± 4.54	71.28 ± 16.69	60.52 ± 14.18
Statistical Analysis F/p Post-Hoc Test Eta Squared (η²) effect size			1.35/0.25	**3.07/0.04** **c > a**,** c > b**	**4.43/0.02** **a > c**,** a > b**	**3.93/0.02** **c > a**,** c > b**
			0.006	0.012	0.025	0.016
**Number of siblings**	0–5	367 (76.5)	92.77 ± 39.28	7.08 ± 5.05	82.56 ± 11.76	58.90 ± 13.81
	6 or more	113 (23.5)	88.01 ± 39.34	6.32 ± 4.63	81.20 ± 13.33	56.39 ± 14.56
Statistical Analysis t/p Hedges’s g effect size			1.12/0.26	1.42/0.15	0.97/0.33	1.66/0.09
			0.121	0,153	0,108	0.176
**Gender**	Female	380 (79.2)	91.85 ± 39.61	6.61 ± 4.97	83.50 ± 10.48	57.56 ± 13.80
	Male	100 (20.8)	90.89 ± 38.31	8.02 ± 4.77	77.48 ± 16.26	61.15 ± 14.52
Statistical Analysis t/p Hedges’s g effect size			0.21/0.82	**-2.53/0.01**	**3.51/0.00**	**-2.28/0.02**
			0,024	0.286	0.505	0.257
**Marital status**	Single	476 (99.2)	91.89 ± 39.26	6.90 ± 4.96	82.35 ± 12.08	58.28 ± 14.03
	Married	4 (0.8)	63.00 ± 39.42	7.75 ± 5.43	69.00 ± 14.44	61.00 ± 13.34
Statistical Analysis t/p Hedges’s g effect size			1.46/0.14	-0.34/0.73	**2.19/0.02**	-0.38/0.70
			0.735	0.171	1.103	0.193
**Income level**	Income is low^a^	252 (52.5)	92.13 ± 38.20	7.36 ± 4.86	82.30 ± 11.17	58.57 ± 14.14
	Equal^b^	202 (42.1)	93.04 ± 40.55	6.35 ± 5.01	82.57 ± 12.49	57.72 ± 13.71
	Goes too much^c^	26 (5.4)	76.11 ± 38.26	6.84 ± 5.33	79.07 ± 17.63	60.34 ± 15.37
Statistical Analysis F/p Eta Squared (η²) effect size			2.18/0.11	2.33/0.09	0.96/0.38	0.49/0.61
			0.009	0.010	0.004	0.002
**Nationality**	Turkish	459 (95.6)	90.99 ± 39.06	6.90 ± 5.02	82.63 ± 11.89	58.34 ± 14.10
	Foreign	21 (4.4)	106.00 ± 42.82	6.95 ± 3.63	73.85 ± 14.82	57.66 ± 12.30
Statistical Analysis t/p Hedges’s g effect size			-1.71/0.08	-0.05/0.95	**2.67/0.01**	0.21/0.82
			0.382	0.010	0.730	0.048
**Maternal education level**	Illiterate^a^	99 (20.6)	84.03 ± 32.68	6.82 ± 5.07	81.88 ± 14.33	60.62 ± 13.96
	Primary school^b^	243 (50.6)	93.18 ± 40.67	7.00 ± 5.03	83.30 ± 10.39	60.04 ± 12.82
	Secondary school^c^	114 (23.8)	94.21 ± 40.10	6.90 ± 4.80	80.80 ± 13.64	58.70 ± 10.71
	University degree^d^	24 (5.0)	95.45 ± 44.77	6.25 ± 4.79	79.83 ± 11.02	56.51 ± 14.67
Statistical Analysis F/p Post-Hoc Test Eta Squared (η²) effect size			2.02/0.11	0.18/0.90	1.58/0.19	**2.84/0.03** **a > d**,** b > d**
			0.010	0.001	0.009	0.017
**Paternal education level**	Illiterate^a^	18 (3.8)	75.11 ± 38.06	8.22 ± 5.34	81.22 ± 17.63	57.61 ± 20.20
	Primary school^b^	204 (42.5)	91.94 ± 40.02	6.58 ± 4.96	83.06 ± 10.91	57.69 ± 14.04
	Secondary school^c^	182 (37.9)	94.11 ± 39.85	7.12 ± 4.90	82.53 ± 12.09	58.95 ± 13.76
	University degree^d^	76 (15.8)	88.89 ± 35.81	6.96 ± 5.05	79.60 ± 13.72	58.60 ± 12.99
Statistical Analysis F/p Eta Squared (η²) effect size			1.43/0.23	0.82/0.48	1.31/0.27	0.27/0.84
			0.009	0.005	0.010	0.002
**Primary place of residence**	Village^a^	69 (14.4)	86.85 ± 40.64	5.79 ± 4.38	83.18 ± 10.48	53.53 ± 13.71
	Town/District^b^	135 (28.1)	94.31 ± 38.63	6.58 ± 4.70	82.62 ± 12.20	57.36 ± 13.69
	City/Metropolitan Area^c^	276 (57.5)	91.54 ± 39.32	7.34 ± 5.18	81.82 ± 12.53	59.97 ± 13.97
Statistical Analysis F/p Post-Hoc Test Eta Squared (η²) effect size			0.82/0.43	**3.44/0.03** **c > a**	0.43/0.64	**6.38/0.00** **c > a**
			0.003	0.013	0.002	0.026
**Current place of residence**	Living with family^a^	240 (50.0)	92.96 ± 39.82	7.17 ± 5.30	82.56 ± 11.51	58.59 ± 14.55
	Private dormitory^b^	18 (3.8)	94.44 ± 47.49	7.22 ± 4.79	76.11 ± 16.49	55.00 ± 13.49
	Public dormitory^c^	222 (46.3)	90.0 ± 38.15	6.59 ± 4.59	82.40 ± 12.35	58.27 ± 13.48
Statistical Analysis F/p Eta Squared (η²) effect size			0.37/0.69	0.80/0.44	1.31/0.27	0.55/0.57
			0.002	0.003	0.010	0.002
**Department**	Nursing	421 (87.8)	92.25 ± 39.28	7.04 ± 4.99	81.87 ± 12.51	58.85 ± 13.77
	Midwifery	59 (12.2)	87.37 ± 39.56	5.93 ± 4.64	84.89 ± 8.78	54.40 ± 15.20
Statistical Analysis t/p Hedges’s g effect size			0.89/0.37	1.61/0.10	**-2.33/0.02**	**2.29/0.02**
			0.124	0.224	0.249	0.318
**Year**	1st year^a^	141 (29.4)	99.18 ± 38.10	6.18 ± 4.72	85.54 ± 8.32	55.58 ± 13.88
	2nd year^b^	143 (29.8)	97.86 ± 37.63	7.34 ± 4.76	80.03 ± 13.28	58.77 ± 13.46
	3rd year^c^	122 (25.4)	89.64 ± 39.42	6.97 ± 4.99	82.52 ± 12.04	57.40 ± 15.19
	4th year^d^	74 (15.4)	84.43 ± 40.09	7.32 ± 5.64	79.78 ± 14.69	61.46 ± 13.54
Statistical Analysis F/p Post-Hoc Test Eta Squared (η²) effect size			**3.71/0.01** **c > b**	1.56/0.19	**7.83/0.00** **a > b**,** a > d**	**4.06/0.00** **c > a**
			0.022	0.010	0.038	0.025
**Do you enjoy your current program of study?**	Yes^a^	239 (49.8)	96.53 ± 39.47	6.13 ± 4.65	83.24 ± 9.76	56.73 ± 13.86
	No^b^	76 (15.8)	84.81 ± 38.77	8.68 ± 5.28	79.10 ± 17.86	63.68 ± 15.25
	Undecided^c^	165 (34.4)	87.72 ± 38.63	7.21 ± 5.03	82.55 ± 11.27	58.12 ± 13.09
Statistical Analysis F/p Post-Hoc Test Eta Squared (η²) effect size			**3.86/0.02** **a > b**,** a > c**	**8.32/0.00** **b > a**	**3.20/0.04** **a > b**	**7.29/0.00** **b > a**,** b > c**
			0.016	0.034	0.013	0.030
**Perceived academic achievement**	Successful^a^	116 (24.2)	97.76 ± 38.74	5.34 ± 4.03	82.61 ± 11.71	56.60 ± 13.96
	Average^b^	341 (71.0)	91.71 ± 38.93	7.25 ± 5.09	82.26 ± 12.09	58.69 ± 13.65
	Unsuccessful^c^	23 (4.8)	59.91 ± 33.44	9.69 ± 5.32	80.21 ± 15.28	61.26 ± 18.79
Statistical Analysis F/p Post-Hoc Test Eta Squared (η²) effect size			**9.20/0.00** **a > c**,** b > c**	**12.04/0.00** **b > a**,** c > a**	0.37/0.68	1.50/0.22
			0.037	0.043	0.002	0.006
**Do you feel your education sufficiently prepares you for your professional life?**	Yes^a^	192 (40.0)	99.89 ± 39.65	5.89 ± 4.56	83.29 ± 9.99	55.18 ± 14.02
	No^b^	126 (26.3)	87.47 ± 42.51	7.88 ± 5.05	79.81 ± 16.40	62.50 ± 14.20
	Undecided^c^	162 (33.8)	85.12 ± 34.50	7.35 ± 5.16	82.89 ± 10.28	58.75 ± 13.00
Statistical Analysis F/p Post-Hoc Test Eta Squared (η²) effect size			**7.58/0.00** **a > b**,** a > c**	**7.32/0.00** **b > a**,** c > a**	**3.50/0.03** **a > b**	**10.90/0.00** **b > a**,** c > a**
			0.030	0.030	0.014	0.044
**Do you feel happy with your life?**	Yes^a^	113 (23.5)	100.38 ± 40.84	4.71 ± 4.15	83.86 ± 8.89	53.46 ± 12.99
	No^b^	112 (23.3)	80.43 ± 39.83	9.91 ± 5.37	77.01 ± 17.69	64.66 ± 14.21
	Somewhat^c^	255 (53.1)	92.70 ± 37.34	6.56 ± 4.47	83.78 ± 10.32	57.67 ± 13.38
Statistical Analysis F/p Post-Hoc Test Eta Squared (η²) effect size			**7.64/0.00** **a > b**,** c > b**	**32.76/0.00** **b > c> a**	**7.67/0.00** **a > b**,** c > b**	**19.98/0.00** **b > c> a**
			0.031	0.134	0.056	0.077
**Are you satisfied with your life?**	Yes^a^	146 (30.4)	101.94 ± 40.25	4.81 ± 3.95	83.76 ± 9.41	53.39 ± 12.77
	No^b^	103 (21.5)	79.95 ± 38.91	9.91 ± 5.53	77.30 ± 17.24	65.03 ± 14.43
	Somewhat^c^	231 (48.1)	90.36 ± 37.34	6.89 ± 4.58	83.33 ± 10.76	58.41 ± 13.35
Statistical Analysis F/p Post-Hoc Test Eta Squared (η²) effect size			**10.05/0.00** **a > b**,** a > c**	**33.55/0.00** **b > c> a**	**6.41/0.00** **a > b**,** c > b**	**22.72/0.00** **b > c> a**
			0.040	0.133	0.046	0.087
**Do you consider living abroad?**	Yes^a^	210 (43.8)	93.14 ± 38.94	7.69 ± 5.19	79.10 ± 15.24	65.00 ± 12.79
	No^b^	145 (30.2)	89.89 ± 40.29	6.05 ± 4.51	85.36 ± 7.77	49.53 ± 13.78
	Undecided^c^	125 (26.0)	91.18 ± 38.99	6.58 ± 4.90	83.92 ± 8.80	57.26 ± 9.77
Statistical Analysis F/p Post-Hoc Test Eta Squared (η²) effect size			0.30/0.73	**5.09/0.00** **a > b**	**12.88/0.00** **b > a**,** c > a**	**67.26/0.00** **a > c> b**
			0.001	0.021	0.054	0.220
**Country (countries) intended for migration** ^*^						
Germany	Yes	129 (26.9)	99.91 ± 37.54	7.41 ± 4.89	81.65 ± 13.50	60.72 ± 12.79
	No	351 (73.1)	89.45 ± 39.52	6.72 ± 4.98	82.46 ± 11.63	57.42 ± 14.35
Statistical Analysis t/p Hedges’s g effect size			**2.38/0.01**	1.34/0.17	-0.65/0.51	**2.29/0.02**
			0.268	0.139	0.066	0.236
United States of America	Yes	101 (21.0)	88.39 ± 38.53	6.99 ± 5.23	80.92 ± 12.62	63.20 ± 12.11
	No	379 (79.0)	92.84 ± 39.58	6.88 ± 4.89	82.60 ± 12.01	57.00 ± 14.21
Statistical Analysis t/p Hedges’s g effect size			-1.10/0.27	0.18/0.85	-1.23/0.21	**4.39/0.00**
			0.113	0.022	0.138	0.449
United Kingdom	Yes	62 (12.9)	88.83 ± 37.03	8.32 ± 5.25	82.09 ± 11.79	62.50 ± 12.94
	No	418 (87.1)	92.06 ± 39.66	8.49 ± 4.89	82.27 ± 12.22	57.69 ± 14.07
Statistical Analysis t/p Hedges’s g effect size			-0.60/0.54	2.41/0.05	-0.10/0.91	**2.53/0.01**
			0.082	0.034	0.014	0.345
Other	Yes	95 (19.8)	91.97 ± 42.06	6.89 ± 5.37	82.77 ± 11.86	60.00 ± 12.98
	No	385 (80.2)	91.57 ± 38.66	6.91 ± 4.86	82.11 ± 12.23	57.89 ± 14.24
Statistical Analysis t/p Hedges’s g effect size			0.09/0.92	-0.03/0.97	0.47/0.63	1.31/0.19
			0.010	0.004	0.054	0.150
**Reasons for wanting to live in another country** ^*^						
Economic and Professional Factors	Yes	195 (40.6)	93.63 ± 38.09	7.73 ± 5.24	79.58 ± 14.43	65.19 ± 12.94
	No	285 (59.4)	90.29 ± 40.13	6.34 ± 4.68	84.06 ± 9.93	53.60 ± 12.73
Statistical Analysis t/p Hedges’s g effect size			0.91/0.36	3.05/0.06	-3.76/0.08	**9.72/0.00**
			0.084	0.269	0.137	0.744
Social and Cultural Factors	Yes	171 (35.6)	92.23 ± 38.96	7.74 ± 5.06	79.45 ± 14.82	62.12 ± 12.72
	No	309 (64.4)	91.32 ± 39.56	6.44 ± 4.85	83.79 ± 10.08	56.20 ± 14.27
Statistical Analysis t/p Hedges’s g effect size			0.24/0.80	**2.75/0.00**	**-3.41/0.00**	**4.52/0.00**
			0.023	0.263	0.362	0.430
Political Factors	Yes	71 (14.8)	91.08 ± 39.40	7.94 ± 4.93	76.21 ± 17.79	63.28 ± 13.81
	No	409 (85.2)	91.75 ± 39.34	6.72 ± 4.95	83.29 ± 10.56	57.44 ± 13.89
Statistical Analysis t/p Hedges’s g effect size			-0.13/0.89	1.90/0.05	**-3.25/0.00**	**3.26/0.00**
			0.017	0.246	0.595	0.420
Personal and Family Factors	Yes	70 (14.6)	95.08 ± 40.22	7.12 ± 4.87	78.47 ± 14.75	61.50 ± 13.21
	No	410 (85.4)	91.06 ± 39.17	6.87 ± 4.98	82.89 ± 11.55	57.76 ± 14.09
Statistical Analysis t/p Hedges’s g effect size			0.79/0.43	0.40/0.68	**-2.38/0.01**	**2.06/0.03**
			0.102	0.050	0.366	0.267
Education-related Factors	Yes	145 (30.2)	95.57 ± 39.53	6.45 ± 4.83	81.69 ± 12.51	60.60 ± 13.10
	No	335 (69.8)	89.95 ± 39.15	7.10 ± 5.01	82.48 ± 12.00	57.32 ± 14.30
Statistical Analysis t/p Hedges’s g effect size			1.43/0.15	-1.31/0.18	-0.65/0.51	**2.36/0.01**
			0.143	0.131	0.064	0.235
Science and Technology-related Factors	Yes	118 (24.6)	98.82 ± 38.83	6.88 ± 4.57	79.49 ± 13.90	60.28 ± 14.18
	No	362 (75.4)	89.31 ± 39.23	6.91 ± 5.09	83.14 ± 11.40	57.66 ± 13.92
Statistical Analysis t/p Hedges’s g effect size			**2.29/0.02**	-0.04/0.96	**-2.58/0.01**	1.76/0.07
			0.243	0.006	0.302	0.187
**Total (**$\overline{X}$±**SD)**			91.65 ± 39.31	10.90 ± 4.96	82.24 ± 12.15	28.31 ± 14.01

^*^Note: Multiple responses allowed

Abbreviations: FESA = Future Expectations Scale for Adolescents; BHS = Beck Hopelessness Scale; RBSA = Religious Belief Scale for Adolescents; ASBD= Attitudes Scale for Brain Drain Among Nursing Students

FESA total scores were significantly higher among first-year students (99.18 ± 38.10), those satisfied with their field of study (96.53 ± 39.47), students who perceived themselves as successful (97.76 ± 38.74), those who considered their education adequate for professional life (99.89 ± 39.65), as well as students who reported being happy (100.38 ± 4.84) and satisfied with life (101.94 ± 40.25). Additionally, students expressing a desire to migrate to Germany (99.91 ± 37.54) and those considering living abroad for scientific and technological reasons (98.82 ± 38.83) also exhibited higher FESA scores (*p* < 0.05) (Table 1).

BHS total scores were significantly higher among participants aged 25 years or older (10.42 ± 4.54), males (8.02 ± 4.77), students who spent most of their lives in urban/metropolitan areas (7.34 ± 5.18), those who enjoyed studying their program (8.68 ± 5.28), those who perceived themselves as unsuccessful (9.69 ± 5.32), those who believed their education was insufficient for professional life (7.88 ± 5.05), and students who reported being unhappy (9.91 ± 5.37) and dissatisfied with life (9.91 ± 5.53). Higher BHS scores were also found among students considering migrating and living abroad (7.69 ± 5.19), particularly for sociocultural reasons (7.74 ± 5.06) (*p* < 0.05) (Table 1).

RBSA total scores were significantly higher among participants aged 18–20 years (83.56 ± 10.38), females (83.50 ± 10.48), single students (82.35 ± 12.08), Turkish nationals (82.63 ± 11.89), midwifery students (84.89 ± 8.78), first-year students (85.54 ± 8.32), those who enjoyed studying their program (83.24 ± 9.76), and students who considered their education adequate for professional life (83.29 ± 9.99). Students reported being happy (83.86 ± 8.89) and satisfied with life (83.76 ± 9.41), those not intending to migrate and live abroad (85.36 ± 7.77), and those not reporting a desire to live in another country due to sociocultural (83.79 ± 10.08), political (83.29 ± 10.56), personal/family (82.89 ± 11.55), or science/technology-related reasons (83.14 ± 11.40) also scored higher on RBSA (*p* < 0.05) (Table 1).

Significantly higher ASBD total scores were observed among participants aged 25 or older (60.52 ± 14.18), males (61.15 ± 14.52), students whose mothers are illiterate (60.62 ± 13.96), those who spent most of their lives in urban/metropolitan areas (59.97 ± 13.97), nursing students (58.85 ± 13.77), fourth-year students (61.46 ± 13.54), students dissatisfied with their program (63.68 ± 15.25), and those who considered their education inadequate for professional life (62.50 ± 14.20). Higher ASBD scores were also observed among students who reported being unhappy (64.66 ± 14.21) and dissatisfied with life (65.03 ± 14.43), those considering migrating and living abroad (65.00 ± 12.79), as well as those intending to migrate to Germany (60.72 ± 12.79), the United States of America (USA) (63.20 ± 12.11), or the United Kingdom (UK) (62.50 ± 12.94). Students who cited economic-professional (65.19 ± 12.94), sociocultural (62.12 ± 12.72), political (63.28 ± 13.81), personal/family (61.50 ± 13.21), or educational reasons (60.60 ± 13.10) for their desire to live abroad also showed significantly higher ASBD scores (*p* < 0.05) (Table 1).

The mean total scores for the scales were as follows: FESA, 91.65 ± 39.31; BHS 10.90 ± 4.96; RBSA, 82.24 ± 12.15; and ASBD, 28.31 ± 14.01 (Table 1).

The FESA total score showed a strong negative correlation with the BHS (*r* = -0.748), a strong positive correlation with the RBSA (*r* = 0.824), and a strong negative correlation with the ASBD (*r* = -0.732) (*p* < 0.05) (Table 2).

**Table 2:** Pearson correlation coefficients among subdimensions and total scores of FESA, BHS, RBSA, and ASBD

Correlations				FESA	BHS	RBSA	ASBD
FESA	Pearson Correlation				**-0.748**	**0.824**	**-0.732**
	Bootstrap^c^	Bias			,002	,000	-,002
		Std. Error			,036	,040	,047
		95% Confidence Interval	Lower		-,878	,750	-,847
			Upper		-,711	,852	-,664
BHS	Pearson Correlation					-0.198	0.203
	Bootstrap^c^	Bias				-,001	,001
		Std. Error				,041	,045
		95% Confidence Interval	Lower			-,275	,117
			Upper			-,112	,287
RBSA	Pearson Correlation						-0.128
	Bootstrap^c^	Bias					,001
		Std. Error					,049
		95% Confidence Interval	Lower				-,217
			Upper				-,024
ASBD							1

Abbreviations: FESA = Future Expectations Scale for Adolescents; BHS = Beck Hopelessness Scale; RBSA = Religious Belief Scale for Adolescents; ASBD= Attitudes Scale for Brain Drain Among Nursing Students; Std. Error (Standard Error) = The standard error of the estimate; it indicates the variability of the statistic across different samples; 95% Confidence Interval = The range within which the true value is expected to fall with 95% probability; Lower = The lower bound of the confidence interval; Upper = The upper bound of the confidence interval

Multiple linear regression analysis revealed that the FESA, BHS, and RBSA scale scores had a significant predictive effect on the ASBD total score. An examination of the model’s coefficients revealed that the FESA total score had a negative and statistically significant effect on the ASBD (β = 0.122, *p* < 0.05). Similarly, the RBSA total score was a negative and significant predictor of the ASBD (β= – 0.098, *p* < 0.05). Conversely, the BHS total score was a positive and significant predictor of the ASBD score (β = 0.226, *p* < 0.001). The tolerance and VIF values ​​of all independent variables in the model indicate that there is no multicollinearity problem. These findings suggest that students’ future expectations, religious belief levels, and hopelessness significantly explain their brain drain attitudes (Table 3).

**Table 3:** Multiple regression coefficients of variables predicting ASBD (FESA, BHS ve RBSA)

Coefficients^a^														
Model		Unstandardized Coefficients		Standardized Coefficients	t	Sig.	95% Confidence Interval for B		Correlations			Collinearity Statistics		Cumulative R²
		B	Std. Error	Beta			Lower Bound	Upper Bound	Zero-order	Partial	Part	Tolerance	VIF	
1	(Constant)	59.247	4.868		12.170	0.000	49.681	68.813						
	FESA (Total)	− 0.644	0.017	0.122	2.580	0.010	− 0.810	− 0.077	− 0.732	− 0.117	0.115	0.876	1.141	0.062
	RBSA (Total)	− 0.114	0.052	− 0.098	-2.171	0.030	− 0.216	− 0.011	− 0.128	− 0.099	− 0.096	0.957	1.045	
	BHS (Total)	0.638	0.136	0.226	4.707	0.000	0.372	0.904	0.203	0.211	0.209	0.855	1.170	

a. Dependent Variable: ASBD (Total)

Abbreviations: FESA = Future Expectations Scale for Adolescents; BHS = Beck Hopelessness Scale; RBSA = Religious Belief Scale for Adolescents; ASBD= Attitudes Scale for Brain Drain Among Nursing Students; B = Unstandardized regression coefficient; Std. Error = Standard error; Beta = Standardized regression coefficient; t = t-statistic; Sig. = Significance value (p-value); 95% Confidence Interval for B: 95% confidence interval for the B coefficient (Lower Bound, Upper Bound); Zero-order = Simple correlation between independent and dependent variable; Partial = Partial correlation; Part = Part correlation; Tolerance = Tolerance (collinearity indicator); VIF = Variance Inflation Factor (collinearity indicator); Cumulative R² = Proportion of variance in the dependent variable explained by all predictors in the model

## Discussion

This study examined the relationships between future expectations, hopelessness, religious beliefs, and migration intentions among nursing and midwifery students at a state university in Gaziantep, Türkiye. The findings indicate that while the overall tendency toward migration was generally low, a considerable number of students are contemplating migration abroad, particularly to Germany, which was the most frequently cited destination. These migration intentions were particularly associated with economic and professional factors. Participants, on average, demonstrated moderate levels of future expectations and hopelessness, along with high levels of religiosity. Notably, students who expressed dissatisfaction with their field of study, perceived their education as inadequate, or reported being unhappy and dissatisfied with life exhibited significantly higher attitudes toward migration. This suggests that although most students are not strongly inclined to migrate, specific subgroups are more vulnerable to emigration-related considerations. To our knowledge, no previous studies have examined the joint relationships among future expectations, hopelessness, religiosity, and migration intentions in this population, highlighting the uniqueness and contribution of the present study.

### Age and academic year

Students aged 25 years or older exhibited higher levels of hopelessness and a stronger tendency toward migration, but lower levels of religious belief. In contrast, first-year students demonstrated more optimistic future expectations and higher religiosity, while fourth-year students showed a more pronounced inclination to migrate. Few studies have directly investigated the relationship between age or academic year and future expectations. The higher future expectations observed among first-year students may be attributed to their lack of exposure to professional challenges and their idealistic outlook. Previous studies reported that hopelessness tends to increase with age [34, 35], and that young students (17 years and older) generally have positive attitudes towards religion [36–38], although some studies found no significant effect of age on religiosity [39]. Likewise, migration tendencies were reported to be higher among individuals under 29 years of age [40] and among fourth-year students [41], while other studies have not found a significant association between age and migration tendencies [42, 43]. As age progresses, it is thought that the responsibilities individuals face, concerns about unemployment, and uncertainties about the future may increase levels of hopelessness and lead to a decrease in spiritual commitment. The lower religiosity observed among students aged 25 years or older may reflect the cumulative effect of stress, disappointment, and life challenges that gradually erode spiritual belief. Conversely, the strong religious beliefs reported by first-year students may serve as a coping mechanism during the transition into academic life, as they grapple with uncertainty and encounter foundational concepts related to health and human existence. Engaging with themes such as life, death, birth, and destiny during early health education may also enhance spiritual awareness. The greater tendency toward migration among older and senior students appears closely linked to growing professional concerns, limited job opportunities, and heightened economic expectations. In this group, a low level of religious belief may be associated with a loss of inner resilience in the face of difficulties, potentially leading individuals to seek solutions abroad. Consequently, a decline in religiosity may indirectly intensify migration intentions.

### Gender differences

In the current study, it was found that female students exhibited higher levels of religiosity compared to male students, while male students showed higher levels of hopelessness and stronger migration tendencies than female students. These findings are consistent with previous research indicating that female students generally hold stronger religious beliefs [44, 45], while male students report higher levels of hopelessness [26, 34] and a greater inclination to migrate [46]. However, the literature also presents contrasting results: some studies have reported stronger religious beliefs among male students [38, 47, 48], higher levels of hopelessness among female students [35, 49], or no significant association between gender and hopelessness [50] or migration tendencies [40, 41, 51]. The stronger religiosity observed among female students may reflect a tendency to seek emotional and spiritual support as a coping strategy. In contrast, the higher migration tendencies among male students may be linked to perceived economic responsibilities and the pursuit of improved career prospects. Additionally, the experience of being in a female-dominated profession such as nursing or midwifery may subject male students to social pressures or professional identity challenges, potentially contributing to increased levels of hopelessness in this group.

### Parental education and family background

Regarding parental education, students whose mothers are illiterate exhibited higher migration tendencies, suggesting that parental educational attainment may indirectly influence individuals’ career and life decisions. Limited maternal education may be associated with lower socioeconomic status and reduced familial support, prompting students to seek improved living conditions and professional opportunities abroad. This inclination appears to be driven not only by the pursuit of personal advancement but also by a desire to provide financial support to their families.

### Program satisfaction, educational perceptions, and migration tendency

In line with the literature findings, students who enjoyed studying their program reported higher future expectations and religious belief levels, whereas those who were dissatisfied with their program showed higher levels of hopelessness and migration tendencies [16, 46, 52, 53]. Individuals who are content with their chosen field of study tend to exhibit greater commitment to their professional goals and draw on their religious beliefs as a source of support amid uncertainty. For these students, religious belief appears to fulfill their search for meaning and help mitigate anxiety about the future. Conversely, students who are dissatisfied with their program may experience reduced professional fulfillment, contributing to diminished hope and heightened feelings of hopelessness. Lower levels of religious belief in this group may limit access to spiritual coping resources, prompting them to seek alternative solutions, such as migrating abroad. These findings underscore the significant role of religious belief in fostering psychological resilience.

### Migration intentions, hopelessness, and religious belief

Among students who reported considering living abroad, higher levels of hopelessness and stronger migration tendencies were observed, whereas those who did not express such intentions demonstrated higher levels of religious belief. The decision to emigrate may stem from various stressors, including language barriers, cultural adaptation, lack of professional recognition, job insecurity, economic uncertainty, and separation from family and social support systems. These challenges can foster a more pessimistic outlook on the future and contribute to heightened hopelessness. In contrast, the elevated levels of religious belief among students who do not consider emigration suggest that faith may help individuals accept their current circumstances and derive meaning from their existing environment. Religious belief may act as an internal stabilizing resource, enabling individuals to interpret adversity through a lens of patience and trust in divine will, thereby reducing the motivation to seek opportunities abroad. In this context, strong religious belief may enhance individuals’ sense of belonging within their society and potentially lessen their inclination to migrate.

### Sociocultural and professional influences on migration

In this study, the finding that nursing and midwifery students who report migration intentions driven by social and cultural factors also exhibit higher levels of hopelessness may reflect the sociocultural challenges they face in Türkiye. Structural problems within the Turkish healthcare system—such as excessive workloads, low wages, and limited opportunities for professional advancement—negatively impact students’ career outlooks and contribute to increased hopelessness. Additionally, traditional gender roles and familial expectations may create further barriers, particularly for female students. Gender-based discrimination and the low societal status of the profession can also adversely affect their psychological well-being. The prevalence of workplace violence, bullying, and psychological pressure in healthcare settings further exacerbates these concerns. Collectively, these sociocultural challenges encountered during the course of their education may foster a pessimistic view of the future and heighten feelings of hopelessness among students.

### Factors discouraging migration: the role of religious belief

In the current study, the finding that students who do not consider emigration due to social and cultural, political, personal and familial, or science- and technology-related factors exhibit higher levels of religious belief may be linked to their sense of stability, belonging, and attachment to traditional values. Individuals with strong religious convictions often tend to uphold existing social structures, maintain close family bonds, and remain rooted in their cultural environment—factors that may discourage them from undertaking major and uncertain life changes such as migration. Emigration entails not only a geographical relocation but also a significant transformation of cultural and value systems, which may be perceived by devout individuals as conflicting with their spiritual beliefs. Consequently, they may prefer to remain within familiar settings. Moreover, religious belief can foster a sense of fate, patience, and acceptance of current circumstances, encouraging individuals to adapt their lives accordingly. Thus, the higher religiosity observed among students not considering migration may reflect both a spiritually grounded sense of belonging and a more cautious attitude toward change.

### Economic, political, and educational factors affecting migration intentions

This study observed that economic-professional, sociocultural, political, personal-familial, and education-related factors may contribute to increased migration tendencies among nursing and midwifery students. Low salaries, difficult working conditions, and limited opportunities for career advancement can drive students to seek countries offering better living standards and professional prospects. A perceived lack of recognition and value for their profession may lead individuals to pursue environments where healthcare workers receive greater societal support. Political instability may further prompt a desire for a safer and more predictable future. Students motivated by the goal of securing improved living conditions for their families and better opportunities for their children may also be more inclined to migrate. Additionally, perceived inadequacies in the educational infrastructure may encourage students to seek higher-quality training and development abroad. Collectively, these factors appear to play a significant role in shaping students’ migration intentions.

### Relationships among future expectations, hopelessness, and religiosity

A strong negative correlation was found between students’ future expectations and their levels of hopelessness. Students with high future expectations tended to possess a positive outlook on their careers and lives, which contributed to greater psychological resilience and reduced hopelessness. This optimistic perspective appeared to buffer against negative emotions and foster emotional well-being. In contrast, students with elevated levels of hopelessness were more likely to adopt a pessimistic stance in the face of uncertainty and adversity, resulting in diminished expectations for the future. These findings underscore the significant influence of individuals’ confidence and hope in shaping their overall life satisfaction and mental well-being.

Students with higher levels of religious belief tended to hold more positive expectations for the future. This association may be attributed to the role of religious belief in providing life with meaning, offering coping mechanisms for uncertainty, and enhancing psychological resilience. Faith systems can help individuals sustain hope in the face of adversity and promote a more optimistic perspective on what lies ahead. Especially for young individuals, religious belief may serve as a psychological anchor during periods of uncertainty, fostering a stronger sense of confidence about the future. These findings suggest that religious belief extends beyond the spiritual domain, exerting a meaningful influence on one’s overall outlook and future orientation. Accordingly, a holistic support approach that addresses students’ spiritual needs may be instrumental in reinforcing their hope and optimism for the future.

### Future expectations as a protective factor against migration

Nursing and midwifery students with higher future expectations were found to exhibit lower tendencies to migrate abroad. This suggests that a strong belief in the possibility of building a fulfilling life and career within one’s own country may reduce the inclination to emigrate. Students who maintain a hopeful outlook toward the future are more likely to perceive adequate opportunities for professional growth and quality of life domestically, which reinforces their willingness to stay. Since migration is often driven by dissatisfaction, high future expectations may serve as a protective factor by mitigating such discontent. These findings highlight the importance of implementing supportive policies that enhance students’ optimism about their prospects and, in turn, help reduce migration tendencies among the healthcare workforce.

## Limitations of the study

This study was conducted with nursing and midwifery students from a public university in Gaziantep. This limits the generalizability of the findings to a broader geographical and demographic context. Additionally, since the Religious Belief Scale (RBSA) used in the study is applicable only to Islam, the results cannot be generalized to different religious or cultural groups. The fact that the study was carried out during a specific period also raises the possibility that temporal factors (e.g., economic crises or global health events) may have influenced the results. These limitations should be taken into account when interpreting the findings and planning future research.

## Conclusion and recommendations

This study, based on a comprehensive analysis of nursing and midwifery students in Gaziantep, revealed how future expectations, hopelessness, and levels of religious belief intersect with tendencies toward migration. The findings show that although a considerable number of students reported considering migration, overall migration tendencies remain relatively low. The most influential factor appears to be a high level of religious belief, which serves not only as a coping mechanism in the face of adversity but also as an internal source of resilience that fosters hope, patience, and a sense of belonging, ultimately reducing migration tendencies. Additionally, as students’ future expectations show a tendency toward increase, their levels of hopelessness and desire to emigrate tend to decrease. Those who feel positively about their profession, consider themselves successful, and perceive their education as adequate are more hopeful about the future and show a lower tendency toward seeking opportunities abroad.

Based on these significant findings, policies targeting healthcare students should adopt a holistic approach that goes beyond economic and academic factors to include psychosocial, professional, and spiritual support. Universities should provide structured academic advising, career counseling, mentorship programs, and internship opportunities to strengthen students’ professional motivation, sense of belonging, and future expectations. Scholarship programs and professional development workshops should be implemented to support students’ educational and career goals, while psychological support services that are sensitive to students’ religious and cultural values should be institutionalized, including seminars, workshops, and counseling that enhance resilience and coping skills through spiritual and cultural frameworks. Furthermore, the quality of education should be continuously improved, and social, economic, and cultural policies should be implemented to increase student satisfaction and professional development opportunities. Support systems that foster hope and achievement should be established, local employment opportunities should be enhanced to reduce migration tendencies and brain drain, and counseling mechanisms should be strengthened to reinforce students’ confidence in building a successful future within their own country. This comprehensive, multi-level approach will not only help prevent brain drain but also strengthen the healthcare workforce and contribute to sustainable social development.

## Data Availability

The datasets generated and/or analyzed during the current study are available from the corresponding author upon reasonable request.
